# Adsorption of Anionic Polyacrylamide onto Coal and Kaolinite Calculated from the Extended DLVO Theory Using the van Oss-Chaudhury-Good Theory

**DOI:** 10.3390/polym10020113

**Published:** 2018-01-25

**Authors:** Wenjie Zou, Jinglin Zhao, Chunbao Sun

**Affiliations:** 1 Civil and Resource Engineering School, University of Science and Technology Beijing, Beijing 100083, China; jlzhaoustb@126.com (J.Z.); suncb@ustb.edu.cn (C.S.); 2 Key Laboratory of Coal Processing and Efficient Utilization, Ministry of Education, Xuzhou 221116, China

**Keywords:** polyacrylamide, coal, kaolinite, extended DLVO theory

## Abstract

The dispersion behavior of particles is of great significance in selective flocculation flotation. The interfacial interaction between coal and the main impurity mineral (kaolinite) particles with the effect of an anionic polyacrylamide (PAM A401) was explored by the extended Derjagin–Landau–Verwey–Overbeek (DLVO) theory. The involved surface free energy components of fine mineral particles were estimated using the van Oss-Chaudhury-Good theory and Washburn equation. After adsorption of PAM A401, the range and absolute value of the hydrophobic interaction *V*_HA_ of the coal particles decreased, the electrostatic repulsive potential increased, and the total potential energy changed from −1.66 × 10^5^ to −4.03 × 10^4^ kT at the separation distance of 5 nm. For interactions between the kaolinite and coal particles after PAM A401 adsorption, the electrostatic repulsive potential increased and the hydrophilic repulsive potential energy decreased. The energy barrier at the separation distance of 0.2 nm decreased from 2.78 × 10^4^ to 2.29 × 10^4^ kT. The total potential energy between the kaolinite and coal particles after PAM A401 adsorption was still repulsive, and the range of the repulsive interaction increased from ~0.05 to 47 nm to ~0.05 to 50 nm. The total potential energy of the coal particles after PAM A401 adsorption was still attractive. This behavior of coal and kaolinite particles with the effect of PAM A401 indicates the possibility of enhanced fine coal separation by the method of selective flocculation flotation.

## 1. Introduction

Polyacrylamides (PAM), a kind of water-soluble polymer with a high molecular weight, are used extensively as flocculants and depressants in filtration, sedimentation, centrifugation, and flotation of mineral processing. Selective flocculation flotation using polyacrylamide is one of the efficient separation methods to separate ultra-fine minerals [1,2,3]. On average, clays account for 60–80% of the total impure minerals in coal [4]. Kaolinite is the most common clay. The adsorption of polyacrylamide onto kaolinite has been attributed to the hydrogen bonding between the silanol and aluminol OH groups at the particles’ surfaces and the polymer’s primary amide functional groups. Electrostatic attraction or repulsion between the charged polyacrylamide and the negatively charged kaolinite surface also makes an effect on the adsorption [5,6,7]. Polymeric flocculants have been reported to adsorb strongly onto coal over shale [8]. The preferential adsorption of polyacrylamides onto the surface of coal was proven to depend on the dosage of the polymer and calgon [9]. The multivalent ions have been indicated to act as a bridge between the negatively charged coal and kaolinite and the anionic groups of the polyacrylamide [10]. The selective adsorption of polymers involving dextrin, guar, and amylose polysaccharide onto hydrophobic solids such as talc and coal is possibly associated with hydrophobic bonding [11,12]. In the coal reverse flotation, it was found that polyacrylamides (molecular weight of 1.5 × 10^6^ g·mol^−1^) with up to 50% degree of anionicity are effective flocculants of fine coal, while conditioning (1500 rev/min) with polyacrylamides of a lower degree of anionicity lead to the selectively flocculated smaller flocs of coal and gangue particles [13]. It was generally confirmed that anionic polyacrylamides with low molecular weights adsorb onto the surface of coal particles selectively compared with kaolinite. Floc size measurements evidenced that the *d*_10_, *d*_50_, and *d*_90_ of coal flocculated by anionic polyacrylamide with a molecular weight of 3 × 10^6^ g·mol^−1^ (PAM A401) with a concentration of 12 mg·L^−1^ at natural pH were 3.18, 2.76, and 2.59 times the corresponding levels of these parameters for kaolinite floc, respectively [14,15].

The fine coal particles being flocculated selectively would promote the recovery of fine combustible material. However, both the wettability of the particles or floc and the interfacial interaction between the particles of the coal slime would be changed due to the effect of PAM. The dispersion behavior of the coal and kaolinite particles with the effect of PAM is another vital factor for the selective flocculation flotation. To date, most of the previous studies on the effect of different polyacrylamides on the separation efficiency of fine minerals and kaolinite flocculation, and the understanding of the interaction mechanisms of anionic polyacrylamide onto coal and kaolinite remain limited.

The interaction energies for the Selective Hydrophobic Coagulation process were appraised using an extended DLVO theory (Derjagin–Landau–Verwey–Overbeek theory) [16]. The adhesion behavior of bacteria onto pyrite was clarified by the extended DLVO theory and surface thermodynamics. Contact angles and zeta potential were measured. The surface energy components were obtained from the contact angle measurements [17]. The extended DLVO theory was also applied to estimate the energy barrier between coal particles and bubbles [18].

The surface thermodynamics of kaolinite and coal proposed by the van Oss-Chaudhury-Good theory have been assessed through the Washburn dynamic method with the flocculation effect of anionic polyacrylamide [14]. The surface free energy components can be obtained by the van Oss-Chaudhury-Good theory as seen in Equations (1) and (2) [19,20]:

(1) $$\gamma_{s}=\gamma_{s}^{\mathrm{LW}}+\gamma_{s}^{\mathrm{AB}}=\gamma_{s}^{\mathrm{LW}}+2{\left(\gamma_{s}^{+}\gamma_{s}^{-}\right)}^{1/2}$$

(2) $$\gamma_{\mathrm{sl}}=\gamma_{s}+\gamma_{l}-2[{\left(\gamma_{s}^{\mathrm{LW}}\gamma_{l}^{\mathrm{LW}}\right)}^{1/2}+{\left(\gamma_{s}^{+}\gamma_{l}^{-}\right)}^{1/2}+{\left(\gamma_{s}^{-}\gamma_{l}^{+}\right)}^{1/2}]$$

where *γ*_s_ (mJ·m^−2^) is the total surface free energy of the solid, *γ*_l_ (mJ·m^−2^) is the surface tension of the liquid, *γ*_sl_ (mJ·m^−2^) is the interfacial free energy of the liquid and the solid, $\gamma_{s}^{-}$ (mJ·m^−2^) is the base part, $\gamma_{s}^{+}$ (mJ·m^−2^) is the acid part, $\gamma_{s}^{\mathrm{AB}}$ (mJ·m^−2^) is the Lewis acid–base, and $\gamma_{s}^{\mathrm{LW}}$ (mJ·m^−2^) is the Lifshitz-van der Waals. AB and LW refer to the polar and nonpolar (dispersion) components, respectively.

In this investigation, subsequent surface energy components and zeta potential change on the mineral surface after adsorption were experimentally determined from the Washburn dynamic method and the mineral zeta potential measurement. An attempt was made to clarify the changes of interaction between coal and kaolinite particles with the flocculation effect of PAM A401 using the extended DLVO theory.

## 2. Experimental

### 2.1. Materials

According to previous work [14,15], PAM A401 was chosen as the polyacrylamide in this investigation. PAM A401 is anionic with an average molecular weight of 3 × 10^6^ g·mol^−1^ and a charge density of 20%. It was provided by Xitao Polymer Co., Ltd., Beijing, China. Kaolinite was taken from Yongcheng, China. The solution of PAM A401 (0.1% *w*/*v*) was prepared using deionized water. It was then diluted to 100 mg·L^−1^.

The coal sample was obtained from a coal preparation plant located in Kailuan, China. The low ash coal sample was separated using a mixed solution (density of 1.3 g·cm^−3^) of benzene and carbon tetrachloride. Benzene and carbon tetrachloride were of AR grade, purchased from Beijing Chemical Works, Beijing, China and used as received. It was washed with deionized water, then dried in a vacuum drying oven (Lichen, Shanghai, China) at 70 °C for 3 h. The low ash coal sample was ground finely (60.45% particles below 20 μm). The ash content was 2.60%.

**Figure 1:**
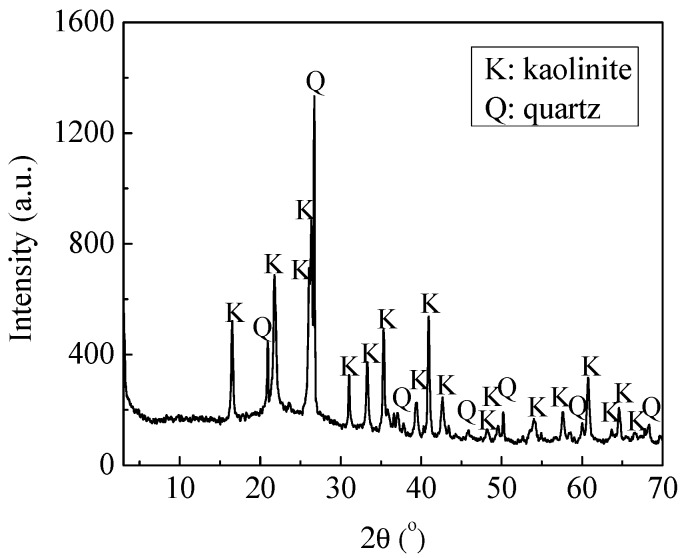
X-ray diffraction pattern of kaolinite.

The X-ray diffraction (XRD) patterns of kaolinite by D8 ADVANCEX (Bruker, Bremen, Germany) are shown in Figure 1. The main mineral was kaolinite. There was a little quartz in the sample. The chemical composition of kaolinite was obtained with the help of S8 Tiger XRF (Bruker, Bremen, Germany). As seen in Table 1, the content of Al_2_O_3_ and SiO_2_ of this sample occupied 92.37%.

Coal and kaolinite samples were shaken for 2 h in a PAM A401 solution of 12 mg·L^−1^ according to the literature [14], and were then dried in a vacuum drying oven at 70 °C for 3 h to obtain the coal and kaolinite adsorbed by PAM A401.

The reagents used in the Washburn dynamic method, including formamide, *n*-hexane, and *α*-bromonaphthalene, were purchased from Sinopharm Chemical Reagent Co., Ltd., (Beijing, China), and were of AR grade.

### 2.2. Methods

#### 2.2.1. Washburn Dynamic Method

The Washburn dynamic method was conducted with the Krüss Tensiometer K100 (Kruss, Hamburg, Germany). The operation sequence is described in detail in the literature [15,21]. Briefly, samples of 2.000 grams were weighed and put into a clean Washburn tube. Filter paper was placed between the tube and sample particle to avoid the particles falling down. To ensure reproducible results, the Washburn tube was lightly hit for the same times with strict uniform force until the sample compaction height was the same for each sample. Then, the Washburn tube was fixed on the hook assembly of the Tensiometer K100. The automatic elevator platform was loaded with a glass beaker filled with no less than 30 mL probe liquid, which rose slowly by ~2 mm from the bottom of the solid samples. As soon as the surface of the liquid touched the bottom of the Washburn tube automatically, the rising of the elevator platform stopped immediately and the Tensiometer K100 started to detect the mass change of the Washburn tube for 200 s to obtain the *ω*^2^-*t* line. In each case, five measurements were conducted for the reproducible result. According to the literature above, the expression for estimating the contact angle can be written as

(3) $$k=c\frac{\rho^{2}\gamma \cos\theta }{2\eta }$$

where *k* is the slope of the *ω*^2^-*t* line, *c* (cm^5^) is the capillary constant of the particle bed, *ρ* (g·mL^−1^) is the liquid density, *γ* (m·Nm^−1^) is the liquid surface tension, *θ* (°) is the contact angle, *η* (mN·m^−^^2^·s^−1^) is the liquid viscosity, *ω* (g) is the liquid weight gain, and *t* is the wetting time (s).

Young Equation, Equations (2) and (3) were combined to get the work of adhesion (*W*_a_), which is:

(4) $$W_{a}=\gamma_{l}(1+\cos\theta )=2[{\left(\gamma_{s}^{\mathrm{LW}}\gamma_{l}^{\mathrm{LW}}\right)}^{1/2}+{\left(\gamma_{s}^{+}\gamma_{l}^{-}\right)}^{1/2}+{\left(\gamma_{s}^{-}\gamma_{l}^{+}\right)}^{1/2}].$$

Assuming that a certain liquid can completely wet the particle bed, the capillary constant of the particle bed (i.e., the parameter *c*) can be obtained using Equation (3). The more completely the liquid wets the particle, the larger is the calculated *c*. One of the four liquids (*n*-hexane, *α*-bromonaphthalene, formamide, and deionized water) was used to determine the capillary constant of the particle bed (i.e., the parameter *c*). The other three liquids were used to determine the advancing contact angle of the mineral particles using Equation (3). The estimated contact angles are reported in Table 2. Then, the component of surface free energy is calculated by Equations (1) and (4).

**Figure 2:**
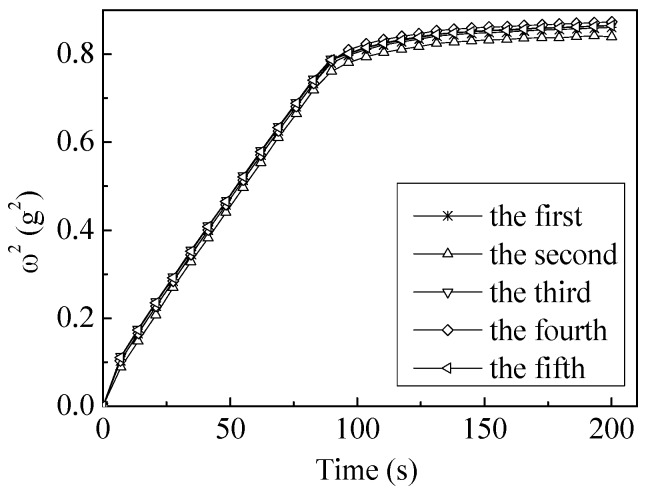
The reproducibility tests for ultra-low ash coal wetted by *n*-hexane.

As an example, five experimental curves of the ultra-low ash coal sample wetting by *n*-hexane are shown in Figure 2. The average slope and standard deviation were 8.23 × 10^−3^ ± 2.7 × 10^−5^.

#### 2.2.2. Zeta Potential Measurement

The ZetaPALS was employed to measure the zeta potential of the coal and kaolinite suspensions. The coal and kaolinite samples were ground to −400 mesh. A suspension containing 0.1 wt % coal or kaolinite was prepared in the deionized water. The prepared suspensions were kept in an ultrasonic bath for 10 min. The PAM A401 solution (100 mg·L^−1^) with a volume of 24 mL was added to the prepared coal or kaolinite suspensions to 0.2 L. After shaking for about 2 h and settling for 15 min in sequence, the upper portion of the suspension was taken for zeta potential distribution measurement. The average of three measurements was used in each case.

## 3. Results and Discussion

### 3.1. Components of the Surface Free Energy

The advancing contact angle of the mineral particles by three kinds of liquid was substituted to Equation (4) to estimate the acid part $\gamma_{s}^{+}$, the base part $\gamma_{s}^{-}$ and the disperse part $\gamma_{s}^{\mathrm{LW}}$. The polar part $\gamma_{s}^{\mathrm{AB}}$ and surface tension *γ*_s_ were obtained by Equation (1). All of the above parameters are collected in Table 3.

The calculated surface free energy components of kaolinite and coal coincided well with the literature [22,23]. Surface free energy components of kaolinite and coal were changed due to the effect of PAM A401.

### 3.2. The Extended DLVO Theory and Calculation of the Relevant Parameters

The total interaction energy between two interfaces *V*_T_ can be written as follows:

(5) $$V_{T}^{}=V_{A}+V_{R}+V_{H}$$

in which *V*_A_ and *V*_R_ are the Lifshitz–van der Waals interaction energy and the electrostatic interaction energy, respectively. *V*_H_ refers to the hydrophobic interaction or hydrophilic repulsive potential energy.

The potential energy between a spherical particle and a larger particle, which may be assumed flat depending on the geometric size ratio, can be calculated by Equations (6)–(8) [24,25,26]:

(6) $$V_{A}=-\frac{AR}{6h}$$

(7) $$V_{R}=\pi \varepsilon R\left(\psi_{01}^{2}+\psi_{02}^{2}\right)\left[\frac{2\psi_{01}\psi_{02}}{\psi_{01}^{2}+\psi_{02}^{2}}\times \ln\left[\frac{1+\exp(-\kappa h)}{1-\exp(-\kappa h)}\right]+\ln\left[1-\exp(-2\kappa h)\right]\right]$$

(8) $$V_{H}=2\pi Rh_{0}V_{H}^{0}\exp\left(\frac{H_{0}-h}{h_{0}}\right)$$

where *A* (J) is the Hamaker constant; *R* (m) is the radius of the spherical particle; *h* (nm) is the separation distance; *ε* (C^2^/(J·m) or F/m) is the dielectric constant of the medium; *ψ*_01_ and *ψ*_02_ (mV) are the Stern potentials of particle 1 and particle 2; *κ^−^*^1^ (m) is the Debye length; *h*_0_ (nm) is the attenuation length, which is between 1 and 10 nm; $V_{H}^{0}$ (mJ/m^2^) is the interaction energy parameter, which is related to the surface wettability; *H*_0_ (nm) is the closest separation distance between two particles.

The Hamaker constant of *A*_132_ is calculated following Equation (9) [27,28]:

(9) $$A_{132}=A_{12}+A_{33}-A_{13}-A_{23}\approx (\sqrt{A_{11}}-\sqrt{A_{33}})(\sqrt{A_{22}}-\sqrt{A_{33}})$$

where *A*_132_ is the Hamaker constant for the particle 1 interacting with particle 2 in a medium 3, *A*_11_ and *A*_22_ are the Hamaker constants for particle 1 and particle 2 in a vacuum, respectively, and *A*_33_ is the Hamaker constant for the medium (water) in a vacuum.

For the Hamaker constant for the substance *i* and *j* in a vacuum (i.e., *A_ij_*), it can be shown that $A_{ij}\approx \sqrt{A_{ii}A_{jj}}$.

In selective flocculation flotation of fine coal, the Hamaker constants are seen in Table 4 and Table 5.

In the calculation of *V*_R_, *ψ*_01_ and *ψ*_02_ are often replaced with the ζ-potential as an approximation. From the zeta potential measurement, the changes are seen in Table 6.

$V_{H}^{0}$ for material 1 interacting with material 2 in a medium 3 can be calculated from Equation (10):

(10) $$V_{H}^{0}=\Delta G_{{131(H}_{0})}^{\mathrm{AB}}=2[\sqrt{\gamma_{3}^{+}}(\sqrt{\gamma_{1}^{-}}+\sqrt{\gamma_{2}^{-}}-\sqrt{\gamma_{3}^{-}})+\sqrt{\gamma_{3}^{-}}(\sqrt{\gamma_{1}^{+}}+\sqrt{\gamma_{2}^{+}}-\sqrt{\gamma_{3}^{+}})-\sqrt{\gamma_{1}^{+}\gamma_{2}^{-}}-\sqrt{\gamma_{1}^{-}\gamma_{2}^{+}}]$$

where $\Delta G_{{131(H}_{0})}^{\mathrm{AB}}$ is the free energy of two surfaces in contact, $\gamma_{i}^{+}$ (mJ·m^−2^) is the acid part, $\gamma_{i}^{-}$ (mJ·m^−2^) is the base part.

Thus, $V_{H}^{0}$ of coal–coal was −3.51 mJ/m^2^, $V_{H}^{0}$ of coal–coal after adsorption of PAM A401 was −0.92 mJ/m^2^, $V_{H}^{0}$ of kaolinite–kaolinite was 45.73 mJ/m^2^, $V_{H}^{0}$ of coal–kaolinite was 16.49 mJ/m^2^, $V_{H}^{0}$ of coal after adsorption of PAM A401–kaolinite was 20.48 mJ/m^2^.

### 3.3. Calculation of the Interaction Energies

(1) Interaction energies between coal particles

**Figure 3:**
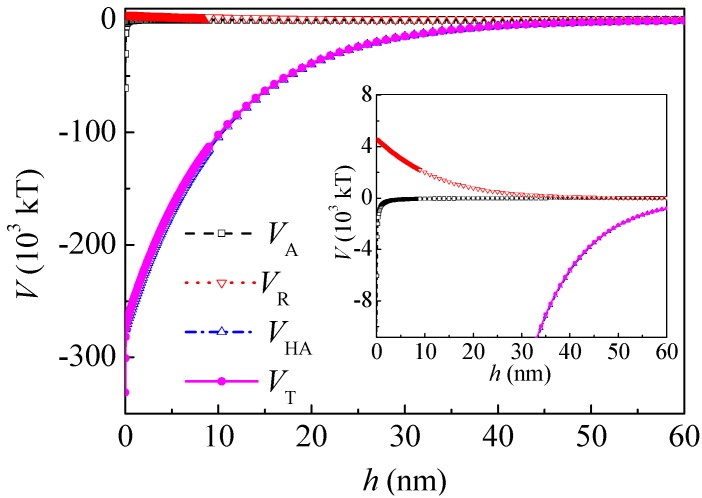
Potential energy between coal particles.

The dielectric constant of water in a vacuum *ε*_0_ = 8.854 × 10^−12^ C^2^/(J·m) and the relative dielectric constant of water *ε*_r_ = 78.5, there is *ε* = 6.95 × 10^−10^ C^2^/(J·m). In a KCl solution (1 mM), there is *κ* = 1.04 × 10^8^ m^−1^ [24]. From the measurement of the zeta potential, *ψ*_01_ = *ψ*_02_ = −20 mV = −0.02 J/C. *R* = 5 × 10^−6^ m, *H*_0_ = 0 nm. According to the literature [24], *h*_0_ = 10.3 nm. From Equations (5)–(8), the curves of the interaction between the coal particles calculated by the extended DLVO theory are shown in Figure 3. The total interaction energy was negative thoroughly in the range of 0–60 nm and minimums occurred, which indicate that the agglomeration of the coal particles was thermodynamically preferred. This coincides well with the literature [16]. The hydrophobic interaction energy *V*_HA_ was about two magnitudes higher than that of the Lifshitz–van der Waals and electrostatic interactions. The *V*_HA_ was relatively long-range, ranging from 0 to 60 nm. When *h* was 5 nm and 20 nm, *V*_HA_ was −1.70 × 10^5^ and −0.395 × 10^5^ kT, and *V*_T_ was −1.66 × 10^5^ and −0.388 × 10^5^ kT, respectively.

After adsorption of PAM A401, *ψ* was substituted by the zeta potential as an approximation *ψ* = −0.029 J/C, and the thickness of the adsorption layer was *δ* = 10 nm according to the literature [21]; therefore, *V*_A_ of the coal particles with PAM A401 layer can be calculated as follows:

(11) $$V_{A}=-\frac{R}{6}\left[\frac{A_{232}}{h}-\frac{2A_{123}}{h+\delta }+\frac{A_{121}}{h+2\delta }\right]$$

**Figure 4:**
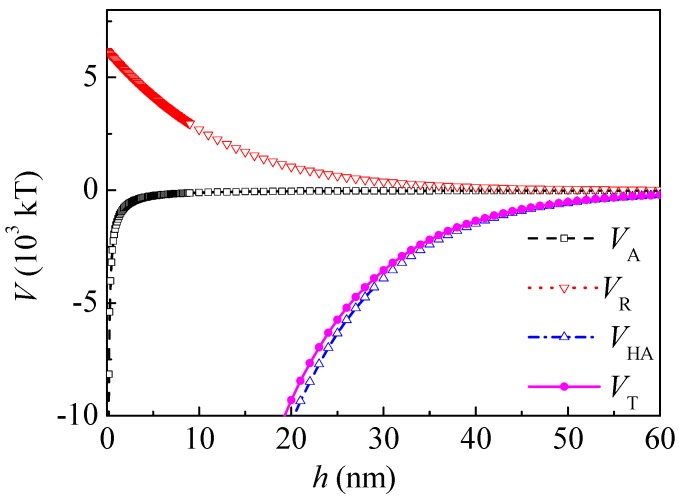
Potential energy between coal particles after adsorption of polyacrylamide A401 (PAM A401).

Figure 4 shows the interaction potential energy between the coal particles after adsorption of PAM A401. At a separation distance *h* of 5 nm and 20 nm, *V*_HA_ was −4.42 × 10^4^ and −1.31 × 10^4^ kT, respectively. The range of *V*_HA_ decreased and *V*_HA_ was about one magnitude higher than that of the Lifshitz–van der Waals and electrostatic interactions. At a separation distance *h* of 5 and 20 nm, *V*_T_ changed to −4.03 × 10^4^ and −0.931 × 10^4^ kT, respectively. The interaction between the coal particles was still attractive in the range of 0–60 nm.

(2) Interaction energies between kaolinite and coal particles

**Figure 5:**
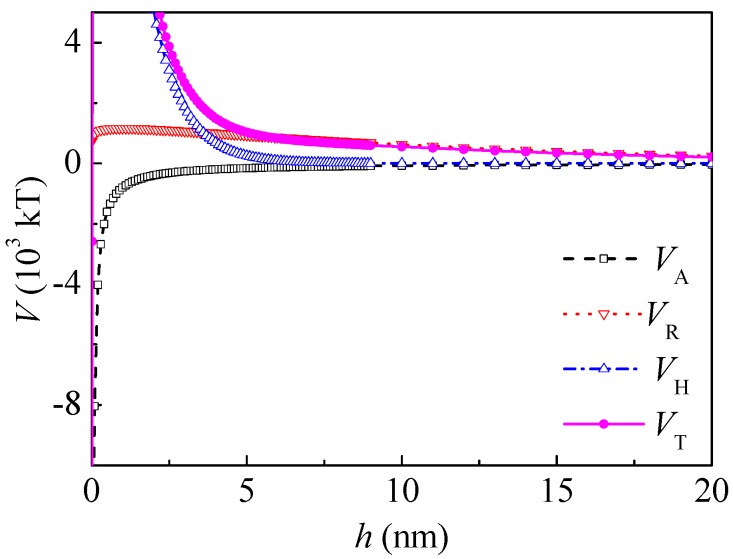
Potential energy between coal and kaolinite particles by extended Derjagin–Landau–Verwey–Overbeek (DLVO).

For kaolinite particles, *R* = 1 × 10^−6^ m. Figure 5 shows the interaction potential energy between coal and kaolinite particles before adsorption of PAM A401.

The total interaction energy was positive at the separation distance ranging from 0.05 to 47 nm, and an energy barrier can be observed, which showed that the total interaction between coal and kaolinite was mainly repulsive. The hydrophilic repulsive potential energy within the range of 0–5 nm dominated the interaction between kaolinite and coal particles. The energy barrier was 2.782 × 10^4^ kT at a separation distance of 0.2 nm. The order of magnitude of the energy barrier was the same with that in the literature [16]. When *h* was 5 and 20 nm, *V*_H_ was 0.25 × 10^3^ and 0 kT and *V*_T_ was 1.03 × 10^3^ and 0.21 × 10^3^ kT, respectively.

**Figure 6:**
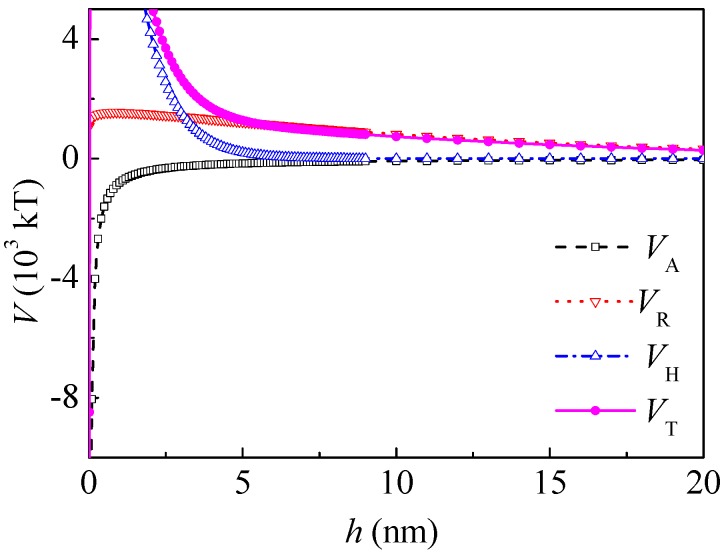
Potential energy between kaolinite and coal particles after adsorption of PAM A401.

According to the literature [14], the adsorption of PAM A401 to kaolinite was much less than that to coal. In order to simplify the calculation, it was assumed that PAM A401 did not adsorb onto the surface of kaolinite. The potential energy between kaolinite and coal-adsorbed PAM A401 is shown in Figure 6. After adsorption of PAM A401, the energy barrier decreased to 2.782 × 10^4^ kT at the separation distance of 0.2 nm. The electrostatic interaction increased and the hydrophilic repulsive potential energy decreased slightly. The total potential energy decreased in the range from 0 to 2.9 nm and increased in the range from 2.9 to 20 nm. When *h* was 5 and 20 nm, *V*_H_ was 0.21 × 10^3^ and 0 kT and *V*_T_ was 1.28 × 10^3^ and 0.28 × 10^3^ kT, respectively. However, the total interaction between kaolinite and coal after adsorption of PAM A401 was still repulsive at a separation distance ranging from 0.05 to 50 nm.

**Figure 7:**
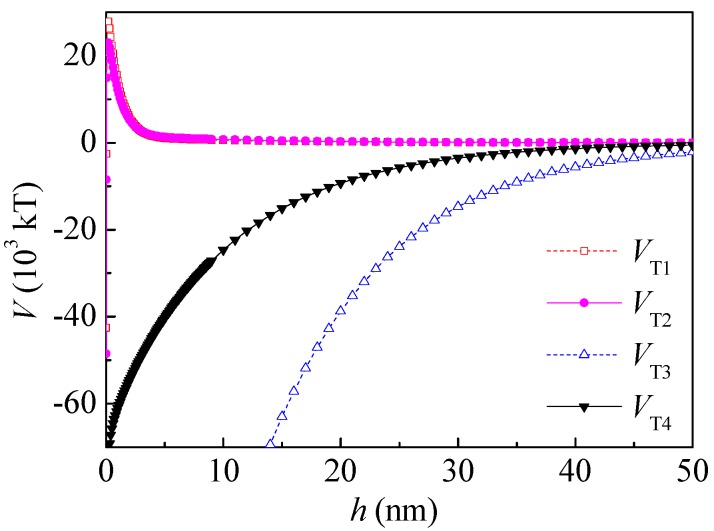
Total potential energy before and after adsorbed PAM A401. *V*_T1_ is the total potential energy between coal particles; *V*_T2_ is the total potential energy between coal particles after adsorption of PAM A401; *V*_T3_ is the total potential energy between kaolinite and coal particles; *V*_T4_ is the total potential energy between kaolinite and coal particles after adsorption of PAM A401.

Therefore, both the surface free energy components and the interaction energies between particles changed after PAM A401 adsorption. As seen in Figure 7, the total potential energy between the kaolinite and coal particles after PAM A401 adsorption was still repulsive, and the range of the repulsive interaction increased from ~0.05 to 47 nm to ~0.05 to 50 nm. The total potential energy of coal particles after PAM A401 adsorption was still attractive. The floc size measured by Microtrac S3500 laser diffractometer (Microtrac Inc., North Largo, FL, USA) evidenced that the *d*_10_, *d*_50_, and *d*_90_ of coal flocculated by PAM A401 with the concentration of 12 mg·L^−1^ at natural pH were 3.18, 2.76, and 2.59 times the corresponding levels of these parameters for kaolinite floc, respectively. The apparent size of the coal particles increased selectively with the bridging effect of flocculants, while the other particles remained in the pulp. Results of the calculation of the interaction energies coincide well with adsorption and flocculation behavior of the coal and kaolinite [14,15,21]. This behavior of coal and kaolinite particles with the effect of PAM A401 demonstrates enhanced recovery of fine coal particles in selective flocculation flotation.

## 4. Summary and Conclusions

The interfacial interaction between particles of kaolinite and coal with the effect of anionic polyacrylamide PAM A401 was explored by the extended DLVO theory. The involved surface free energy components of fine mineral particles were determined using zeta potential measurements, the van Oss-Chaudhury-Good theory, and the Washburn equation. It was shown that both the surface free energy components and the interaction energies between particles changed. After adsorption of PAM A401, the total potential energy of the coal particles changed from −1.66 × 10^5^ to −4.03 × 10^4^ kT at the separation distance of 5 nm. For interactions between kaolinite and coal particles after PAM A401 adsorption, the energy barrier at the separation distance of 0.2 nm decreased from 2.78 × 10^4^ to 2.29 × 10^4^ kT. The total potential energy between the kaolinite and coal particles after PAM A401 adsorption was still repulsive, and that of the coal particles after PAM A401 adsorption remained attractive. The apparent size of the coal particles increased selectively with the bridging effect of flocculants, while the other particles remained in the pulp. This behavior of coal and kaolinite particles with the effect of PAM A401 is of great significance in selective flocculation flotation of fine coal.

## Figures and Tables

**Table 1:** Chemical composition of kaolinite by XRF.

Chemical Composition	Al_2_O_3_	SiO_2_	Fe_2_O_3_	CaO	MgO	Na_2_O	K_2_O	Ti_2_O	P_2_O_5_
Content (%)	45.84	46.53	0.31	0.16	0.068	0.23	1.09	0.86	0.16

**Table 2:** The estimated contact angles.

Sample	*n*-Hexane	*α*-Bromonaphthalene	Formamide	Deionized Water
	Contact Angle θ (°)			
Coal	0	40.80	63.92	82.90
Kaolinite	43.76	0.17	9.28	0
Coal adsorbed PAM A401 (12 mg/L)	0	44.60	58.35	76.30
Kaolinite adsorbed PAM A401 (12 mg/L)	33.99	0	18.49	14.76

**Table 3:** Surface free energy components of kaolinite and coal with the effect of PAM A401 (at 20 °C).

Samples	$\gamma_{s}^{\mathrm{LW}}$	$\gamma_{s}^{+}$	$\gamma_{s}^{-}$	$\gamma_{s}^{\mathrm{AB}}$	$\gamma_{s}$
Coal	32.28	0.62	23.46	7.64	39.92
Kaolinite	44.40	0.39	58.27	9.53	53.93
Coal after adsorption	30.54	0.98	24.93	9.89	40.43
Kaolinite after adsorption	44.40	0.32	55.05	8.34	52.74

**Table 4:** The Hamaker constants of materials in a vacuum.

Material	*A* (kT)
air	0
water	8.98
coal	14.73
kaolinite	75.24
PAM [29]	19.42

**Table 5:** The Hamaker constants of two materials in water.

Particle 1	Medium 3	Material 2	Hamaker Constant (kT)
coal	water	air	−2.55
coal	water	coal	0.73
coal	water	kaolinite	4.83
kaolinite	water	air	−17.01
kaolinite	water	kaolinite	32.23

**Table 6:** Zeta potential of coal and kaolinite with adsorption of PAM A401 (12 mg/L).

Sample	Zeta Potential (mV)	
	Before Adsorption	After Adsorption
Coal	−20 ± 0.62	−29 ± 0.67
Kaolinite	−45 ± 0.76	--
